# Fever, pulmonary interstitial fibrosis, and hepatomegaly in a 15-year-old boy with Gaucher disease: a case report

**DOI:** 10.1186/s13256-018-1848-z

**Published:** 2018-10-21

**Authors:** Meng Yang

**Affiliations:** The First People’s Hospital of Yunnan Province, 157#, Jinbi Road, Kunming City, Yunnan Province China

**Keywords:** Gaucher disease (GD), Splenectomy, Pulmonary interstitial fibrosis

## Abstract

**Background:**

Gaucher disease is an autosomal recessive disorder resulting from the accumulation of glucocerebroside in the cells of the macrophage-monocyte system caused by deficiency in lysosomal glucocerebrosidase. Intravenously administered enzyme replacement therapy is the first-line therapy for Gaucher disease type 1 and substrate reduction therapy represents an alternative oral treatment. Here is a rare case report of Gaucher disease in South China.

**Case presentation:**

Our patient was a 15-year-old Han Chinese boy presenting with fever, edema, and gradually increasing abdominal girth. A physical examination revealed obvious hypoevolutism and hepatomegaly, and laboratory tests and imaging examinations showed severe pulmonary interstitial fibrosis, infection, and moderate anemia. A final diagnosis of Gaucher disease was confirmed after examining the splenic pathological section derived from a splenectomy performed 6 years ago. His recovery improved after receiving anti-infection, diuresis, blood transfusion, and hepatoprotection and so on. However, enzyme replacement therapy was not adopted by our patient because his family could not afford it.

**Conclusion:**

A rare case of Gaucher disease is reported here to emphasize the importance of early recognition by clinical manifestation and histological findings. Gaucher disease should be considered in the differential diagnosis of children with unexplained symptoms of multiple systems.

## Background

Gaucher disease (GD) was initially described by Philippe Gaucher more than a century ago in his doctoral thesis in 1882, when he reported the infiltration of enlarged cells in a spleen. The biochemical basis for GD was elaborated 83 years later (1965) by Roscoe Brady’s group. The molecular basis of the disease was elucidated in the late 1980s, when the glucocerebrosidase gene mutations were identified. There are three clinical subtypes, which are delineated by the absence or presence and progression of neurologic involvement: type 1 or the non-neuronopathic form; type 2, the infantile-onset, acute neuronopathic form; and type 3, the juvenile-onset neuronopathic form. All three subtypes are inherited as autosomal recessive traits, of which type 1 disease is the most common. GD has a higher incidence at birth among Ashkenazi Jews of approximately 1 in 450. The worldwide incidence is between 1:40,000 and 1:86,000, but in South China, the morbidity is approximately 1 in a million.

GD is a lysosomal storage disease caused by autosomal recessive mutations in the glucocerebrosidase gene, *GBA1*, encoding acid beta-glucosidase, and is one of the most common lipid storage diseases (LSDs). Causal gene defects in GD lead to impaired intracellular lipid balance in cells of monocyte/macrophage lineage, and subsequent infiltration of the liver, spleen, bone marrow, and other tissues with lipid-laden macrophages. Typical clinical manifestations include hypoevolutism, hepatosplenomegaly, anemia, thrombocytopenia, spontaneous fracture, and neurological signs.

GD can be diagnosed through enzyme activity assay (EAA), molecular diagnosis, bone marrow aspiration, and pathological biopsy. Treatment modalities for GD include enzyme replacement treatment (ERT), substrate reduction therapy (SRT), bone marrow transplantation, splenectomy, and blood transfusion in selected cases. ERT is considered first-line therapy, and lifelong treatment has proven effective therapeutic efficacy.

The aim is to describe a 15-year-old boy with non-consanguineous parents who had a history of splenectomization, suffered from pyrexia of unknown origin, and equivocal investigations. Raising awareness of early diagnosis of such cases and consideration of unusual symptoms can have a positive impact on the progress and long-term management.

## Case presentation

Our patient was a 15-year-old Han Chinese boy who presented with fever, weakness, pallor, edema, dyspnea, and gradually increasing abdominal girth for 2 weeks. His parents denied consanguineous marriage. His medical history was not significant. His one older sister and two younger sisters were all healthy. He was delivered after full-term normal pregnancy. His growth and development were markedly slow after birth, but his school records were excellent. His spleen was resected due to unexplained enlargement at the age of 9. He had a history of multiple prolonged hospital admissions for pyrexia of unknown origin, without reaching a clear diagnosis.

**Fig. 1:**
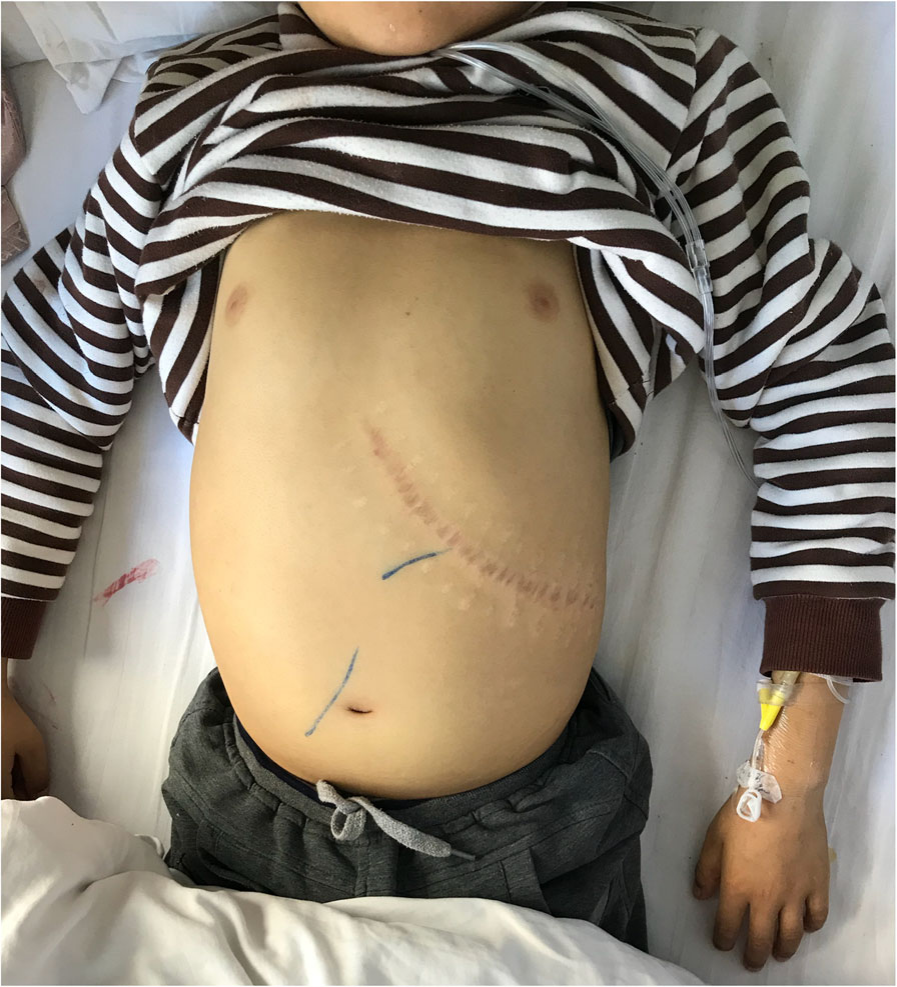
Abdominal distention, a previous surgical scar located in the left upper quadrant, and the liver expanded 10 cm below costal margin

The results of an initial assessment on admission were: he looked unwell, height 135 cm, weight 30 kg, and no sign of secondary sex characters. He had fever with a temperature fluctuating between 37.2 and 39.7 °C, pallor, wakefulness, distress, and clarity of mind. There was no jaundice, petechiae, or fresh rash on skin. Superficial lymph nodes were palpable in inguinal region. His eyelids were swollen, no cyanosis, pharynx without congestion, and bilateral tonsils III° large. There was no thyroid enlargement. Trachea in midline. There were audible harsh breath sounds and crackles bilaterally. His heart beat was 114/minute with normal rhythm, no murmur was heard. He had a distended abdomen with hepatomegaly; liver palpable 10 cm below costal margin with firm consistency and free from tenderness. He had a huge scar located in the left upper quadrant (Fig. 1). Murphy sign (−); shifting dullness sign (+). There was marked pitting edema in his lower extremities. A neurological assessment was normal. The results of other systemic examinations were essentially normal.

**Fig. 2:**
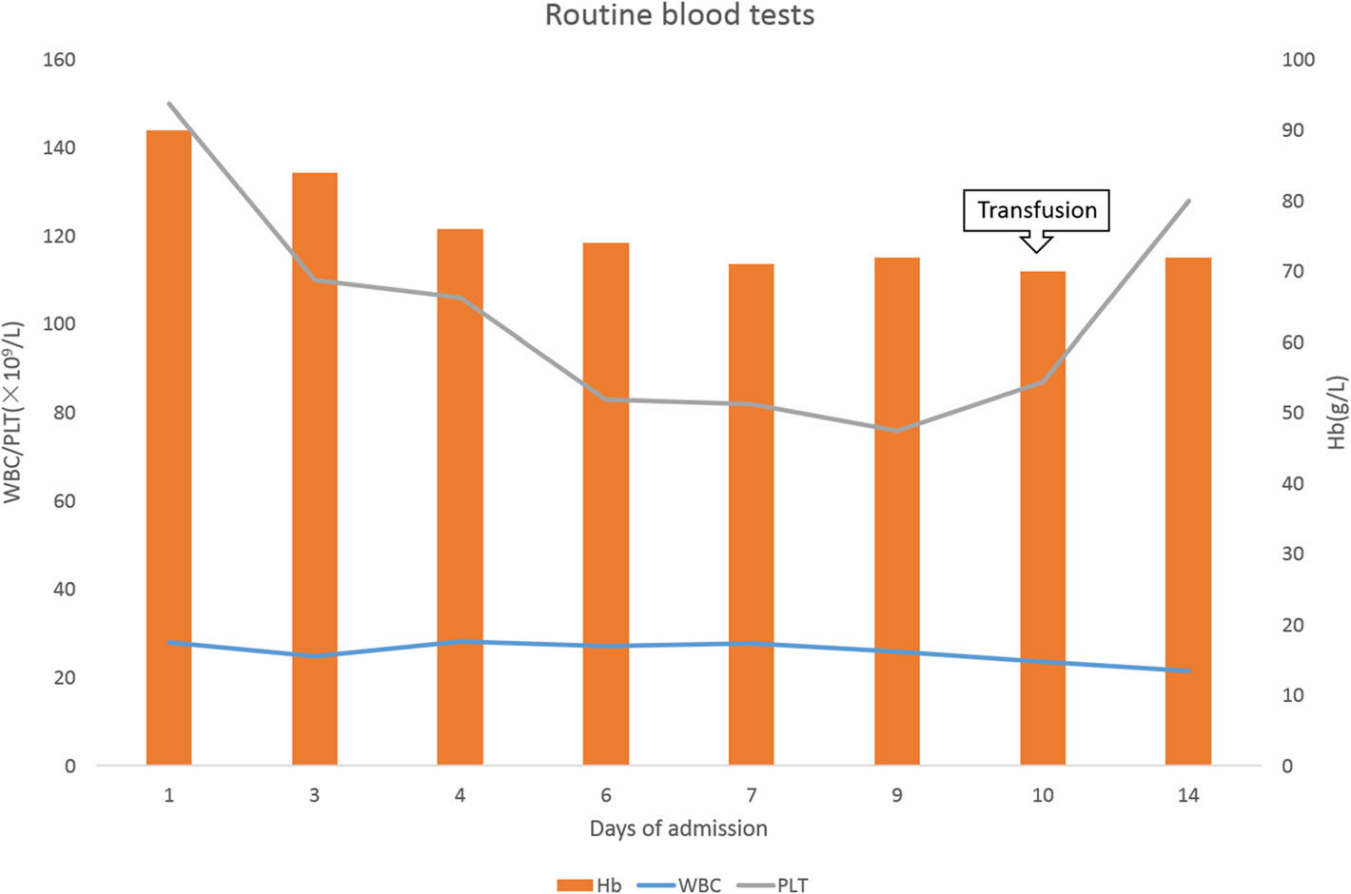
Changes of main indexes of blood routine tests during hospitalization. *Hb* hemoglobin, *PLT* platelets, *WBC* white blood cell

**Fig. 3:**
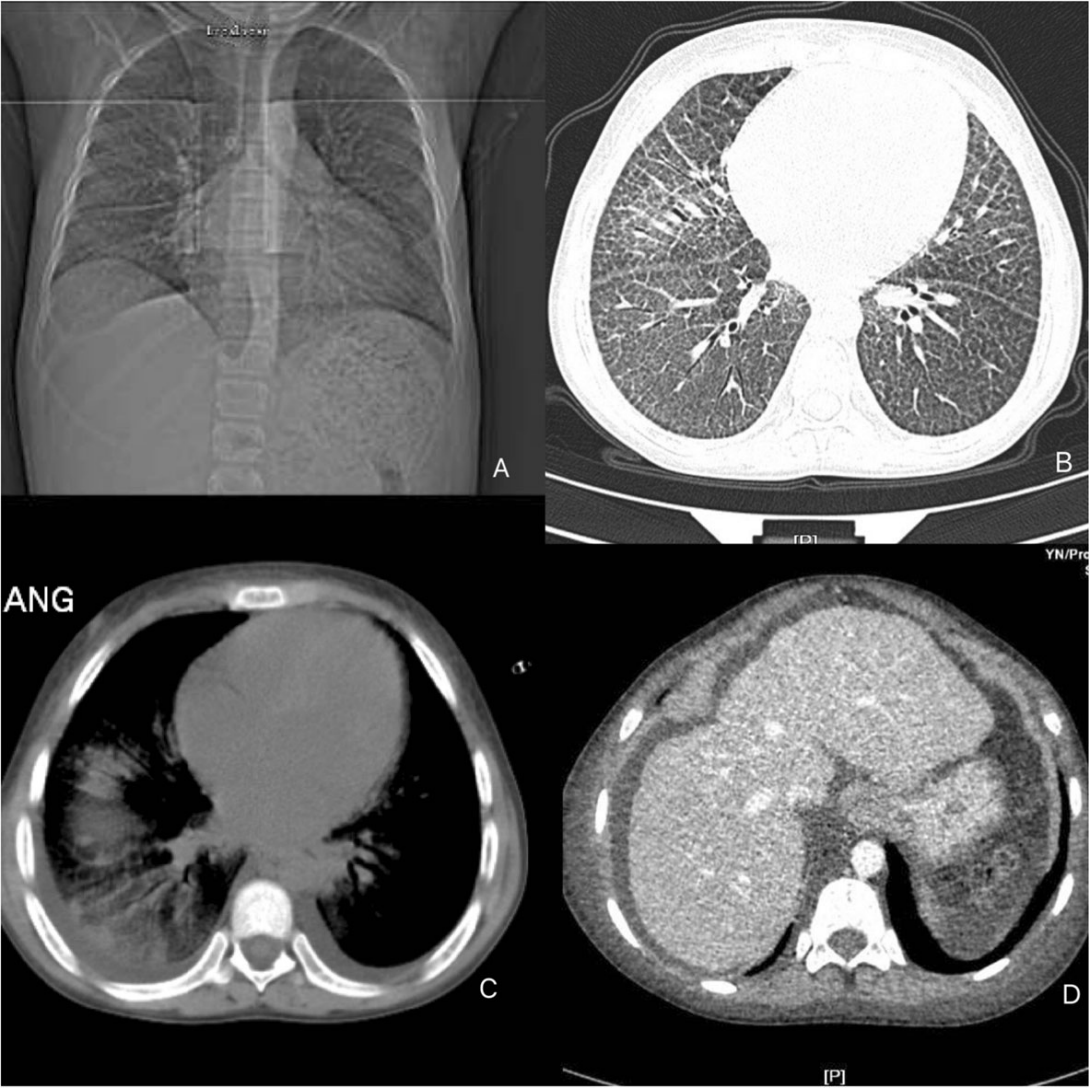
**a** Anteroposterior chest X-ray showing a bilateral reticulonodular interstitial pattern. **b** Computed tomography pulmonary window showing bilateral reticular change, ground-glass opacity of the lung parenchyma, characteristic of the paving pattern, and pulmonary interstitial fibrosis. **c** Computed tomography mediastinal window showing exudation, consolidation, and a small amount of pleural effusion. **d** Enlarged liver with irregular shape

Initial and subsequent laboratory tests and imaging revealed the following results:

1. On routine blood tests conducted over several days, white blood cell (WBC) count was more than 20 × 10^9^/L with normal classification, whereas hemoglobin (Hb) and platelets (PLT) decreased continuously (Fig. 2). Liver enzymes and bilirubin were increased slightly to moderately with hypoproteinemia: lactate dehydrogenase (LDH) 301 U/L, aspartate transaminase (AST) 45 U/L, alkaline phosphatase (ALP) 218 U/L, total bilirubin (TBIL) 72.9umol/L, albumin (ALB) 29 g/L, but a kidney function test and urine analysis were unremarkable. Prothrombin time (PT) and activated partial thromboplastin time (APTT) were prolonged slightly; D-Dimer increased moderately.
2. Other positive indicators were: procalcitonin (PCT) fluctuated around 0.2 ng/ml; antistreptolysin (ASO) was 259 IU/mL; Widal’s test TH 1:160, TO 1:320; tumor marker CA-25 was 384.0 U/mL, CA724 was 38.78 U/mL; ferritin was 550.58 ng/mL; hepatitis E virus antibody IgG(+); and *Legionella pneumophila* serum antibody IgM(±). The tests of thyroid function, erythrocyte sedimentation rate (ESR), C-reactive protein (CRP), CD64 index, T-lymphocyte subsets, and glucose-6-phosphate dehydrogenase (G6PD) were normal. Coombs test, interferon-γ release assay, antinuclear antibodies (ANAs), hepatitis A virus (HAV)\hepatitis B virus (HBV)\hepatitis C virus (HCV)\human immunodeficiency virus (HIV) antibody test, and herpes simplex virus (HSV)\cytomegalovirus (CMV)\Epstein–Barr virus (EBV)-deoxyribonucleic acid (DNA) test were negative.
3. Blood and fungal cultures were negative. Bone marrow smear showed infectious bone marrow image and occasional atypical lymphocytes.
4. Ultrasound revealed grossly enlarged liver (right oblique diameter 164 mm) with normal echotexture, gallbladder wall swelling, left renal calculi, massive peritoneal effusion, but portal vein and common bile duct had normal diameters. There was slight pericardial effusion with normal pulmonary arterial pressure. Enhanced computed tomography (CT) scans showed severe pulmonary interstitial fibrosis with infection, multiple lymph node display, liver shape irregularity, and no obvious abnormality in abdominal angiography (Fig. 3).

**Fig. 4:**
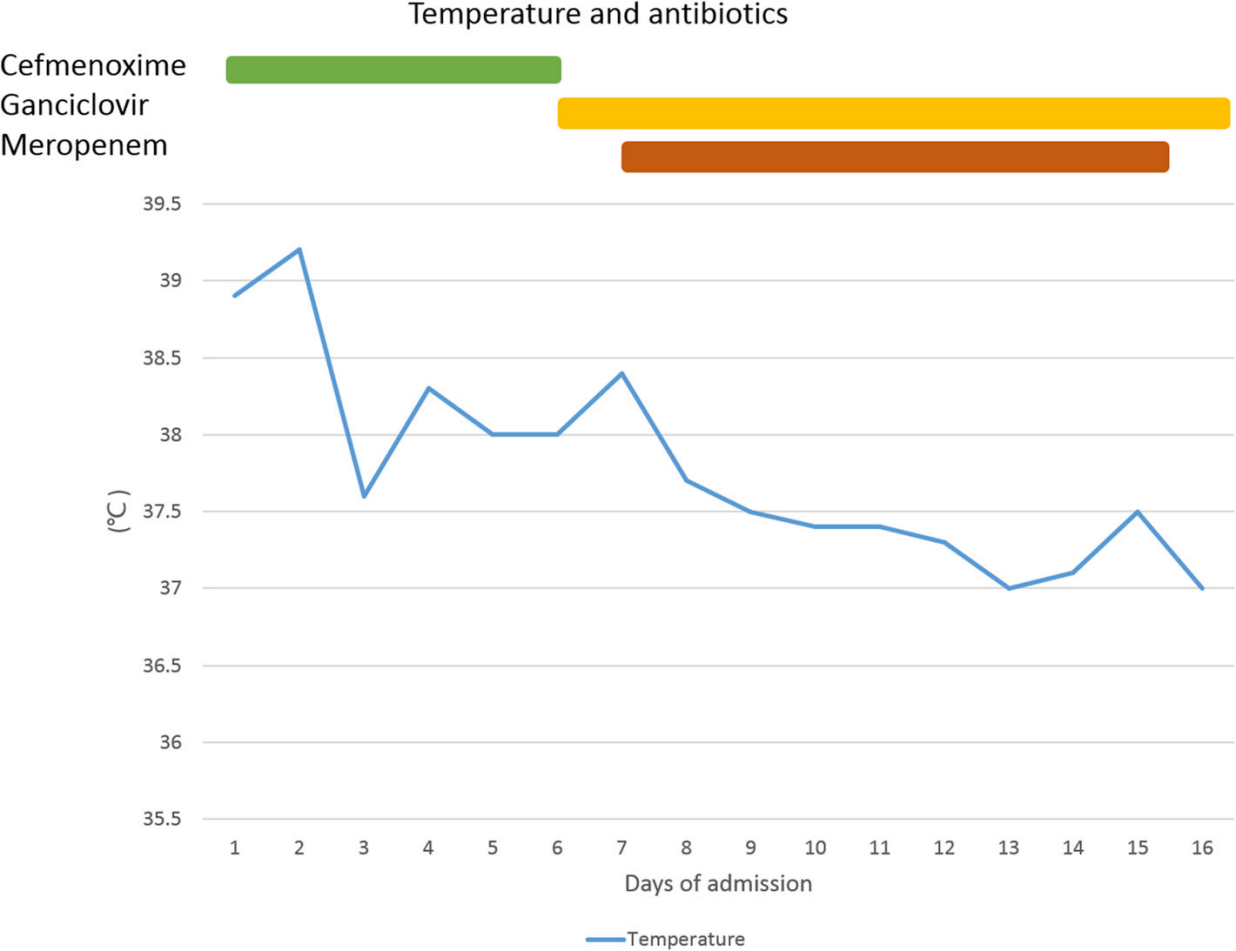
Temperature relative to antibiotic therapies. Colored bars represent treatment timings in relation to *x*-axis time after treatment start

After receiving therapies including anti-infection, anti-virus, atomization inhalation, diuresis to eliminate swelling, liver-preserving and gallbladder protection, human serum ALB, immunoglobulin and blood transfusion, the symptoms of our patient improved, and his peak body temperature decreased (Fig. 4). However, it was difficult to explain the illness with “one etiology”; it was assumed that it was probably a kind of rare genetic metabolic disease associated with infection. To confirm the diagnosis, pathological sections of the spleen were borrowed from another hospital, where the boy underwent splenectomy 6 years previously.

**Fig. 5:**
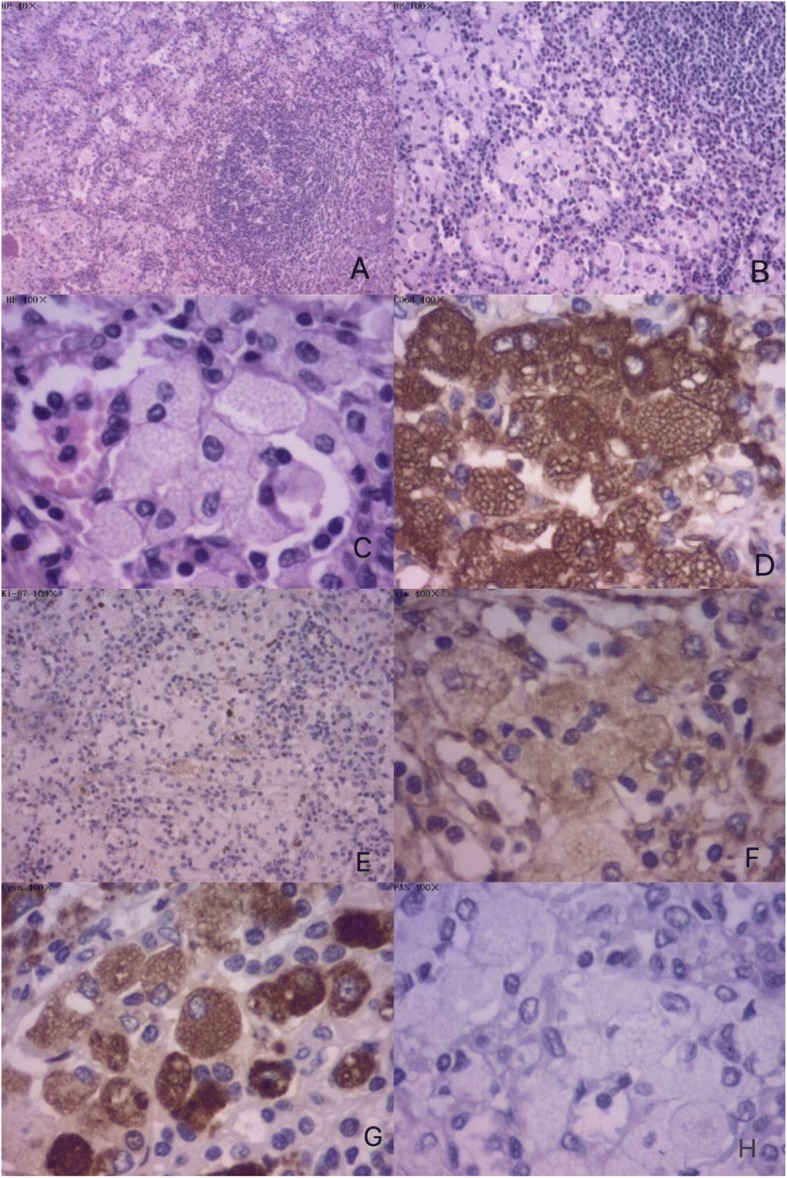
Microscopic examination of the spleen. **a** Splenic trabecula disappeared, and some splenic nodules remained. **b** Red pulp was occupied by a large number of Gaucher cells. **c** Higher power field (400×) exhibiting diffuse infiltration by foamy macrophages with vacuolated cytoplasm (Gaucher cells). **d** Immunohistochemical stain CD68 (+). CD68 denotes histocyte. **e** Lyso (+). Lyso also denotes histiocytic source. **f** Vim (+). Vim hints at mesenchymal source. (**g**). Ki-67 (+<5%) indicates proliferative activity. **h** Specific stain periodic acid–Schiff (−) ruling out mucus

Hematoxylin-eosin (HE) staining of the spleen sections showed that red pulp was occupied by a large number of Gaucher cells, splenic trabecula disappeared, and some splenic nodules remained. The results of specific stains were: Rf (+) and periodic acid–Schiff (PAS) (−). The results of immunohistochemical stains were: CD68 (+), Lyso (+), Vim (+), KI-67 (+<5%), Pan-CK (−), EMA (−), HHF35 (−), CD31 (−), CD34 (−), LCA (−), and S-100 (−) (Fig. 5). Final diagnosis was GD (type 1). However, our patient’s family declined to test beta-glucosidase levels, gene mutation site, or purchase imiglucerase (enzyme replacement therapy). Finally, he was discharged from hospital after his temperature had declined to normal.

During a subsequent 6-month telephone follow-up, he said that he was able to continue with his school life, and do some mild physical activities; he no longer had a fever. A routine blood examination at a local hospital showed mild anemia, and a CT scan showed no significant progress of pulmonary infection. After raising enough money, his family would consider using imiglucerase.

## Discussion

This is a rare case of GD which has a very low morbidity of approximately 1/1,000,000 in China. Due to China’s huge population base, the number of patients with GD is estimated to be more than 1000 nationwide, but it is rarely diagnosed and reported internationally. The characteristics of this case are a long course of disease, a difficult diagnosis, multisystem involvement, a poor prognosis, and it involves social economics. The diagnosis and treatment of this disease involves pediatrics, genetic metabolism, internal medicine, imaging, pathology, molecular biology, and so on. There are few reports in the Gaucher literature regarding intractable cases, standardization of the radiological presentations, and supportive treatment. The existing case reports mainly focus on the efficacy of ERT. This report aims to emphasize the importance of early recognition by clinical manifestation and histological findings. GD should be considered in the differential diagnosis of children with unexplained symptoms in multiple systems.

GD has an autosomal recessive pattern of inheritance and is caused by glucocerebrosidase deficiency; glucocerebrosides cannot be hydrolyzed to glucose and N-acyl-sphingosine, resulting in the accumulation in macrophages of the reticuloendothelial system. Macrophages that have thus been altered are referred to as Gaucher cells [1]. Glucocerebrosidase is encoded by the human *GBA1* gene. The *GBA1* gene is located on chromosome 1q2. There are over 300 known mutations that can cause GD, the most common mutations are c.1226A>G (N370S), 84GG, IVS2+1, and the c.1448T>C (L444P). The L444P mutation homozygous state has a very high association with neuropathic variants of GD [2]. Molecular studies for mutation are beneficial for confirming diagnosis, screening family members, and formulating the prognosis [3].

The accumulation of Gaucher cells in various tissues leads to damage of multiple organ systems. GD should be considered in children of all ages presenting with visceromegaly, recurrent infections, bone pain, fatigue, and thrombocytopenia. Accumulation of glucocerebrosides in liver and spleen causes hepatosplenomegaly and hypersplenism. Splenomegaly is the most typical presenting sign of the non-neuronopathic form; hepatomegaly is universal but does not have the same magnitude as splenomegaly. Hepatic fibrosis, cirrhosis, and portal hypertension are uncommon unless the patients have prior splenectomy during childhood. In the lungs, it can lead to repeated pulmonary infections; in the bone marrow, it can affect hematopoietic function and cause bone destruction (osteoporosis, frequently). In the nervous system, it can result in growth retardation and corresponding nerve dysfunction [4, 5].

GD is the most prevalent lysosomal storage disease and is traditionally classified into three major phenotypes: type 1 (the chronic, non-neuropathic, adult type), which accounts for 99% of all cases and is characterized by a clinical profile with little clinical evidence; type 2 (the acute, neuropathic, infantile type), which usually results in death before the age of 2 due to pneumonia and anoxia; and type 3 (the subacute, neuropathic, juvenile type) is characterized primarily by epileptic seizures that start at around age 10 and has a poor prognosis. Other less prevalent types are the perinatal-lethal and cardiovascular forms.

Diagnostic delays are not unusual. A preliminary diagnosis can be made on the basis of clinical manifestations, but a definitive diagnosis should be based on beta-glucocerebrosidase activity or DNA detection [6]:

1. EAA: measurement of acid beta-glucosidase activity in peripheral blood leukocytes, urine, or cultured skin fibroblast. Glucosidase activity of less than 15% is diagnostic. The EAA is sensitive, specific, and much less invasive; it can be carried out prenatally.
2. Molecular diagnosis: DNA analysis of the *GBA* gene for the four most common mutations accounts for most instances of the disease. Gene mutation study on N370S, L444P, 84GG, and IVS2, which can be carried out prenatally, account for approximately 90% of the disease-causing alleles.
3. Bone marrow aspiration: aggregation of large Gaucher cells. It is not necessary to establish a diagnosis as Gaucher cells can be sparsely distributed and give false negative result. Pseudo-Gaucher cells could be found in the marrow of some patients with chronic myeloid leukemia (CML), type II congenital dyserythropoietic anemia, thalassemia, Hodgkin lymphoma, multiple myeloma, and acquired immunodeficiency syndrome (AIDS).
4. Other tests: biopsy of the spleen or other tissues, peripheral smear, liver function test, brainstem-evoked potential, ultrasound of the abdomen, chitotriosidase level test, and MRI or CT scan of the chest, bone, liver, and spleen, and so on [7, 8].

Presently, ERT is a very expensive and lifelong treatment and should be the main method for any therapeutic plan. Intravenously administered ERT with recombinant glucocerebrosidase is given every 2 weeks at a high dose. It is effective in reducing the liver and spleen size, and ameliorating skeletal abnormalities and hematological abnormalities of disorder, although lung manifestation may not respond satisfactorily to ERT. Our patient is unable to purchase ERT because it is very expensive. SRT inhibits glucosylceramide synthase that catalyzes the first step in the biosynthesis of glucosylceramide and subsequently reduces the biosynthesis of more complex glycosphingolipids. It is indicated in patients who are unsuitable for ERT [9]. Bone marrow transplantation may be considered when ERT is not feasible, but is rarely performed due to a high risk of significant complications and mortality. Splenectomy may be required on rare occasions, infection with encapsulated bacteria is a complication, and other manifestations of GD, such as increasing glycolipid deposition in the lung and bone marrow, are well-known adverse effects of splenectomy. Blood transfusion is indicated in some patients with anemia. The goal is to increase Hb level to 110 g/L or more in children with GD. Coexisting etiologies for anemia must always be evaluated and ruled out [8].

Pulmonary involvement is frequently identified in GD; however, there are no epidemiological studies of the issue yet. In the literature, there is a lack of standardization of the radiological presentations of GD, due to the multifactorial involvement with multiple patterns of tissue infiltration by Gaucher cells. The imaging characteristics of GD correspond to several pathophysiological mechanisms. In addition to thickening of the interlobular and intralobular septa, patients with GD can present with alveolar opacities, capillary plugging by Gaucher cells, and interstitial opacities, with a predominance of lymphatic distribution, as well as respiratory infections. Other alterations described include pulmonary fibrosis, a miliary pattern, and involvement of the hilar or mediastinal lymph nodes, as well as a reduction in lung volume as a consequence of hepatosplenomegaly. Radiographic examinations can reveal an interstitial pattern and can show changes in bone structures [10, 11].

The diffuse pulmonary involvement seen in patients with GD indicates that it is a systemic disease. CT is an important tool for the initial evaluation and follow-up of these patients, and lung biopsy can be dispensed with when the tomography reveals interstitial opacities in an appropriate clinical and epidemiological context. When there is no clinical suspicion of GD, a tomographic finding of the crazy-paving pattern makes the radiologic diagnosis difficult. In such cases, the main differential diagnoses are alveolar proteinosis, pulmonary hemorrhage, pulmonary vasculitis, diffuse alveolar damage (acute respiratory distress syndrome), pulmonary edema, bronchioloalveolar carcinoma, Niemann–Pick disease, and radiation pneumonitis, as well as viral, lipoid, mycobacterial, interstitial, eosinophilic and *Pneumocystis carinii* pneumonia [12].

Our patient’s GD was type 1, the initial symptoms were unexplained hypersplenism and splenomegaly, which can cause mechanical compression of lung tissue and lead to lung disease [13]. Although pulmonary symptoms were not so obvious and lung function was not significantly affected, pathological changes in his lungs were visible because of the depositions of Gaucher cells. According to an autopsy report by Lee and Yousem [14], 30% of patients with GD have obvious pathological lung changes, such as: (1) Gaucher cell deposition causing fibrosis around bronchi, capillaries, and alveoli; (2) pulmonary hypertension due to Gaucher cell deposition and pressure on pulmonary capillaries; and (3) chronic inflammatory invasion of pulmonary alveoli. Because the disease progression is variable, corresponding signs and symptoms vary and may include pulmonary hypertension, pulmonary interstitial lesions, and hypoxemia (mostly due to increased lung shunting). The present studies indicate that the immune system of patients with GD is defective. Undoubtedly, depositions of Gaucher cells in lung tissue cause pathological changes and deficient immune cell function so that patients are susceptible to pulmonary infections [15].

According to the guidelines [16, 17], cephalosporins are preferred for treatment of community acquired pneumonia, therefore, we chose to initiate treatment with cefmenoxime empirically. A chest radiograph showed bilateral lung involvement, and lesions had expanded slightly compared with a radiograph of days before, and our patient’s oxygen index was normal. These findings were consistent with the clinical manifestations of severe pneumonia. Therefore, we used combination therapy with meropenem (plus cefmenoxime) in treatment of Gram-positive bacteria, Gram-negative bacteria, and anaerobes. However, his condition continued to worsen. The antiviral drug ganciclovir was added and human serum ALB and immunoglobulin were administered to enhance his immunity. After several days, his temperature gradually returned to normal, leukocyte and neutrophil counts declined slowly, and chest radiography findings improved. We considered that the pulmonary infection might have been caused by conditional pathogenic bacteria and viruses.

For 50% of patients who develop infections after splenectomy, the pathogenic bacterium is pneumococcus [18]. Therefore, the possibility of a mixed postoperative infection could not be excluded. Regarding immune support treatment, human immunoglobulin by intravenous injection is a kind of passive immunotherapy, which can increase the immunoglobulins level, enhance the patient’s antibody response, neutralize toxins directly, and kill both bacteria and viruses, resulting in immunity enhancement of the patient [19]. Because immunity declines after splenectomy, increasing patients’ susceptibility to severe infections, it is important to provide immune support treatment. However, because of our patient’s economic situation, we did not administer early immune support treatment and only resorted to this when the infection became severe and antibiotics were ineffective.

Based on this case, we can draw the conclusion that the administration of immune support treatment, such as intravenously administered human immunoglobulin, could be effective for preventing infections. Dynamic monitoring of pulmonary function, such as blood tests, etiological detection, and chest radiography, will lead to timely detection and, therefore, timely treatment of any infections. In the event of a lung infection, wide-spectrum antibiotics should be administered empirically, with subsequent adjustment of antibiotics based on the results of cultures. While awaiting culture results, human immunoglobulin should be administered to enhance passive or active immunity. In addition, attention must be paid to basic monitoring and treatment measures, such as hepatic/renal function, coagulation function, water and electrolyte balance, and nutritional support. All these are critical for maintaining patients’ vital signs and other physiological parameters in the normal or near-normal range.

## Conclusions

GD should be considered in the differential diagnosis of patients with unexplained splenomegaly and symptoms in multiple systems especially with an extended period of time. Recurrent pulmonary infection needs attention and active treatment. Moreover, the early recognition of GD would lead to safe and effective treatment that could decrease morbidity and reduce as far as possible visceral and skeletal involvement.
